# Prediction of drug–target interactions through multi-task learning

**DOI:** 10.1038/s41598-022-23203-y

**Published:** 2022-10-31

**Authors:** Chaeyoung Moon, Dongsup Kim

**Affiliations:** Department of Bio and Brain Engineering, Korea Advanced Institute of Science and Technology (KAIST), Daejeon, 34141 Republic of Korea

**Keywords:** Drug discovery, Cheminformatics, Machine learning

## Abstract

Identifying the binding between the target proteins and molecules is essential in drug discovery. The multi-task learning method has been introduced to facilitate knowledge sharing among tasks when the amount of information for each task is small. However, multi-task learning sometimes worsens the overall performance or generates a trade-off between individual task’s performance. In this study, we propose a general multi-task learning scheme that not only increases the average performance but also minimizes individual performance degradation, through group selection and knowledge distillation. The groups are selected on the basis of chemical similarity between ligand sets of targets, and the similar targets in the same groups are trained together. During training, we apply knowledge distillation with teacher annealing. The multi-task learning models are guided by the predictions of the single-task learning models. This method results in higher average performance than that from single-task learning and classic multi-task learning. Further analysis reveals that multi-task learning is particularly effective for low performance tasks, and knowledge distillation helps the model avoid the degradation in individual task performance in multi-task learning.

## Introduction

Drug discovery requires enormous time and cost, but has a poor success rate^1,2^. Identifying suitable molecules with the desired activity from a chemical space composed of more than 10^60^ molecules is difficult^3^. Because drugs work by binding target proteins associated with a disease, molecules are screened to find those that bind a target protein and show the desired activity in early drug discovery^4,5^. This process usually requires substantial time and cost. Therefore, computational modeling for predicting molecular activity on targets has been developed to enable higher efficacy and lower cost in this process^6^.

Quantitative structure–activity relationship, or QSAR is a method for identifying relationships between molecular structure and biological activity^7,8^. Machine learning methods, such as random forest, have been applied to QSAR modeling^7,9^. Recently, deep learning has shown promising results in various fields, such as computer vision^10^, natural language processing^11^, and games^12^. Hence, studies have incorporated deep learning into drug discovery, such as prediction of binding affinity or ADME-Tox properties^13–18^ and prediction of drug–target interaction or drug–drug interaction^19–23^.

Generating a well-trained model requires a large amount of data. One of the major difficulties in using molecular data is the insufficient amount of labeled data. Substantial time and expense are required to produce molecular data from experiments^7^. Consequently, multi-task learning has been suggested to address this problem^16^. Multi-task learning refers to training multiple tasks simultaneously with a single model. In a multi-task neural network, information or knowledge regarding different tasks is shared through the weight-shared layers. Therefore, multi-task learning benefits the training process through data amplification and eavesdropping^24,25^. Accordingly, the multi-task model has been applied to QSAR and has led to improved performance over the single-task model^13–18^.

Although multi-task learning can leverage performance through knowledge sharing, it sometimes worsens the performance or generates a trade-off between individual task performance and the average task performance. Thus, many studies have examined how to select the tasks trained together in multi-task learning^26,27^. In QSAR modeling, one study has shown the importance of selecting similar tasks in a multi-task model to obtain a performance gain^18^. The authors have found that using a multi-task model for diverse targets worsens rather than improves the model’s performance. However, performance has been improved with a multi-task model for targets with similar binding site sequences^18^. Additionally, another study has shown that the gain from the multi-task learning in QSAR modeling tends to occur when molecules in the datasets of tasks are similar, and the tasks are correlated^28^.

Another possible method to overcome the worse performance of multi-task learning than single-task learning is knowledge distillation. Knowledge distillation is a training method that transfers the knowledge from a “teacher” model to a “student” model by guiding the student model to follow the predictions of the teacher model^29^. Knowledge distillation has been used for compressing models. Recent studies have shown that this method also makes the student model outperform its teacher^30,31^. In a recent study^31^, the authors present a training method called Born-Again Multi-tasking (BAM) to address the performance degradation of multi-task learning. They apply knowledge distillation to a model of natural language understanding tasks through a novel method called teacher annealing. In this method, the rate of predictions of the teacher model gradually decreases while the rate of the true label increases during training. Consequently, the BAM model outperforms both single-task learning and multi-task learning models. Inspired by these studies, we propose a method that benefits from the advantage of multi-task learning and minimizes possible negative effects. We apply group selection and knowledge distillation in the multi-task learning setting to molecular binding prediction tasks. In molecular binding prediction, molecules are regarded as data samples, and their binding targets are regarded as tasks. Therefore, targets are grouped according to their similarities in group selection. We use a ligand-based similarity approach to determine the similarity between targets. Similarity ensemble approach or SEA^32,33^ is applied for selecting similar targets. SEA is a method that forms associations between targets according to their active ligand set similarity. It computes the ligand similarity based on ligand structure and uses it to estimate the similarity between targets. This method has been successfully used in research associated with the molecule-target binding task. Similarity between targets are computed by SEA and hierarchical clustering is applied to group the similar targets. The more details of our group selection using SEA are explained in Methods: target clustering section.

For knowledge distillation, we use the method similar to BAM. First, we train the models by single-task learning, and then train the models by multi-task learning. During training, we apply knowledge distillation, such that the multi-task learning models are guided by the predictions of the single-task learning models. As in^31^, the teacher annealing method is also applied. Combining this group selection and knowledge distillation allows the model to obtain an average performance increase with less of an individual performance decrease, thereby improving performance in molecular binding prediction over that of single-task learning and classic multi-task learning.

This study comprises three experiments. In the first experiment, we report that classic multi-task learning involving training all targets together results in poorer performance of molecular binding prediction than the single-task learning model. In the second experiment, we show that applying group selection to multi-task learning improves model performance. In the final experiment, we demonstrate that combining group selection and knowledge distillation results in better performance than applying only group selection. Further analysis shows that the tasks benefit more from multi-task learning when their initial performances from single-task learning are lower. This finding suggests that our multi-task learning method can be effectively used when its predictive power toward a specific target is particularly lower than that toward others. In addition, the gain from knowledge distillation increases with increasing performance loss after application of multi-task learning. This result indicates that introducing knowledge distillation to a multi-task learning helps the model restore its individual task performance.

## Results and discussion

### Target clustering based on SEA

To compute the similarity between targets, we use SEA. The threshold value for the raw score is determined to be 0.74. Similarities between targets are calculated by using this threshold. Then similar targets are grouped into clusters on the basis of the SEA result for multi-task learning. Table 1 shows a summary of clustering results. The largest cluster has 11 targets, and the smallest has two targets. The number of clusters is 103, and overall, 268 targets are included in these clusters. Details of the clustering results are shown in Supplementary Table S1 online.

**Table 1:** Target protein clustering results.

Cluster size	Cluster counts
11	1
6	2
5	3
4	8
3	20
2	69

There are total of 103 clusters and 268 targets.

### Multi-task learning on entire targets

We first build a single-task model for QSAR modeling whose task is defined as predicting molecular binding to a specific target, and then apply multi-task learning using entire 268 targets. The model is tested on the test set of every target and calculates an AUC score for each target (target-AUROC) to produce 268 target-AUROC values. We use threefold cross-validation and held-out test set for model evaluation. For threefold cross-validation, we repeat the train and test process three times to test the model on every fold. The reported evaluation value is the average of three values. For evaluation on held-out test set, we run the models with five different random seeds: 0, 1, 2, 3 and 4. Every target has five target-AUROC values, and the average of these five values is reported. The results shown in Table 2. indicate that multi-task learning on 268 targets results in lower average performance than that of single-task learning. The mean target-AUROC over 268 targets are 0.709 and 0.690 for single-task learning and multi-task learning, respectively. The mean target-AUPRC over 268 targets of single-task learning is 0.825 and the mean accuracy over 268 targets is 0.749. The mean target-AUPRC over 268 targets of multi-task learning is 0.811 and the mean accuracy is 0.746. We calculate the robustness, which is defined as the proportion of tasks for which the target AUROC is higher than that of a single task learning model. The robustness of the multi-task learning on entire targets is 37.7%, meaning that target-AUROC decreases in 61.6% of tasks.

**Table 2:** Comparison of the single-task learning model results and results of the two multi-task learning models.

			Single-task learning	Multi-task learning (entire)	Multi-task learning (clustered)
Held-out Test dataset	AUROC	Mean	0.709	0.690	**0.719**
		Std	0.183	0.155	0.172
	AUPRC	Mean	0.825	0.811	**0.832**
		Std	0.216	0.221	0.212
	Accuracy	Mean	0.749	0.746	**0.753**
		Std	0.216	0.208	0.212
Threefold Cross-validation	AUROC	Mean	0.908	0.895	**0.909**
		Std	0.063	0.071	0.062
	AUPRC	Mean	**0.956**	0.951	**0.956**
		Std	0.068	0.073	0.067
	Accuracy	Mean	**0.884**	0.881	**0.884**
		Std	0.077	0.079	0.079

The mean value is the average target-AUROC, target-AUPRC, target-accuracy over 268 targets.

Significant values are in bold.

**Figure 1:**
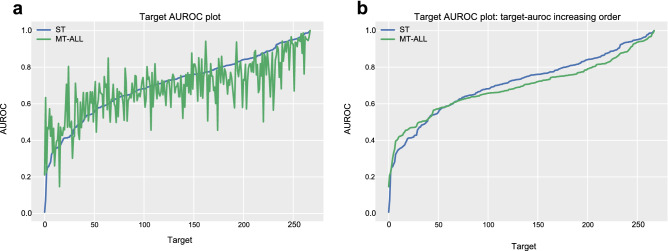
AUROC plot for a single-task learning model and multitask learning model. The blue line is a plot for the single-task learning model (ST), and the green line is for the multi-task learning model (MT-ALL; multi-task learning on entire targets). (**a)** The target order is the same in the two plots. (**b)** The target order of the two plots differs. The targets are ordered in target-AUROC increasing order.

Figure 1 shows the AUROC of every target resulting from the single-task learning model and the multi-task learning model. We order the targets on the basis of their AUROC from the single-task learning model in the left figure (Fig. 1a). To compare the target-AUROC distribution of two models, we order the targets in target-AUROC ascending order in the right figure (Fig. 1b). In the right figure (Fig. 1b), the plot of the multi-task learning model is located higher than the plot of the single-task learning model in the area where the AUROC is between approximately 0.25 and 0.6. The ratio of targets with relatively low target-AUROC is lower in the multi-task learning model than the single-task learning model. However, the area where the AUROC is above 0.6 decreases in the target-AUROCs in many tasks. Wilcoxon signed-rank test^34^ with the scipy^35^ library confirms that applying multi-task learning to similar tasks significantly decreases the performance (p-value < 0.0006).

### Multi-task learning on similar targets

After clustering the targets by similarity, we apply multi-task learning to the clusters of similar targets. Then we compare the results with those from the single-task learning model and the previous multi-task learning model, with all tasks trained in the same model. Table 2 shows the prediction results of the single-task neural network and two multi-task models. The first is the model training all tasks together by one model, and the other is the model training similar tasks included in each cluster. The single-task learning model results show that the mean target-AUROC over 268 targets is 0.709, and the standard deviation is 0.183. Mean AUPRC over 268 targets is 0.825 and mean accuracy is 0.749. The multi-task learning model results show that the mean target-AUROC is 0.719, and the standard deviation is 0.172 when the model is trained on similar targets. The mean AUPRC over 268 targets is 0.832 and mean accuracy is 0.753. From the result table, the multi-task learning model shows a higher performance than that of the single-task learning model. Among 268 targets, the AUROC improves in 157 targets, representing 58.6% of the overall targets in clustered multi-task learning. The threefold cross-validation results are consistent with held-out test dataset results.

**Figure 2:**
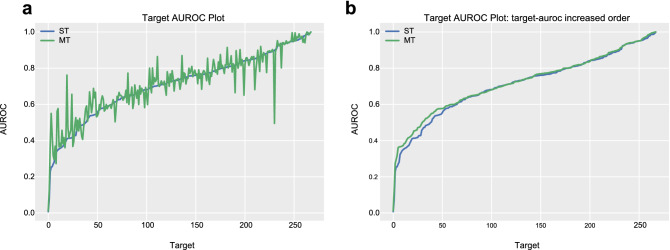
AUROC plot for the single-task learning model and multi-task learning model on similar targets. The blue line is a plot for the single-task learning model (ST), and the green line is a plot for the multi-task learning model (MT). (**a)** The target order is the same in the two plots, (**b)** the target orders of the two plots differ. The targets are sorted in target-AUROC increasing order.

Figure 2 shows the AUROC of every target resulting from the single-task learning model and the multi-task learning model. The targets are ordered according to their AUROC from the single-task learning model in the left figure (Fig. 2a). To compare the target-AUROC distribution of the two models, we order the targets in target-AUROC ascending order in the right figure (Fig. 2b). In the right figure (Fig. 2b), the plot of the multi-task learning model is located higher than the plot of the single-task learning model in the area where the AUROC is between approximately 0.25 and 0.6. The ratio of targets with relatively low target-AUROC is lower in the multi-task learning model than the single-task learning model. Wilcoxon signed-rank test^34^ confirms that applying multi-task learning to similar tasks significantly improves the performance (p-value < 0.001).

We further compute the correlation between the target-AUROC of the single-task learning model and the target-AUROC difference (see Supplementary Fig. S1 online). The target-AUROC difference is calculated by subtraction of the target-AUROC of the single-task learning model from the target-AUROC of the multi-task learning model. The positive target-AUROC difference indicates that this task benefits from multi-task learning with similar targets. The pearsonr function from scipy^35^ is used in the Pearson correlation test. The Pearson correlation coefficient is −0.337, and its p-value is 1.498e−08. The result supports the conclusion that tasks with lower performances benefit more from multi-task learning with similar targets.

### Multi-task learning with knowledge distillation

As discussed in the previous section, multi-task learning with similar tasks increases the performance over that of standard single-task learning. However, in some individual tasks, the performances decrease after multi-task learning. Therefore, we apply the knowledge distillation method to minimize this problem. We investigate whether knowledge distillation is beneficial for predicting molecular binding tasks, especially by reducing the number of tasks with decreased performance. We train the two different multi-task learning with knowledge distillation models: one with single-task learning model as the teacher denoted as ST → MT and the other with multi-task learning model as the teacher (MT → MT). We also test the single-task learning model distilled by the single-task model (ST → ST) to determine the effect of knowledge distillation on single-task learning.

Table 3 shows the results of the three models. As explained in the previous section, both threefold cross-validation and testing on held-out test set are used for evaluation. The reported value in held-out test dataset results is the average results over five times and the reported value in threefold cross-validation is the average results over three times. Among the three models, the multi-task learning model distilled by the single-task learning model (ST → MT) shows the highest mean performance. The performance of the multi-task learning model increases after application of knowledge distillation. Both multi-task learning models distilled by single-task learning and multi-task learning models show higher mean performance than those without distillation. In contrast, the performance of the single-task learning model decreases after knowledge distillation. However, the threefold cross-validation results are not consistent with the held-out test set results. Learning without knowledge distillation results show the higher mean performance than learning with distillation.

**Table 3:** Comparison of the various knowledge distillation model results.

			ST → ST	ST → MT	MT → MT
Held-out Test dataset	AUROC	Mean	0.708	**0.722**	0.720
		Std	0.182	0.174	0.173
	AUPRC	Mean	0.825	**0.834**	0.833
		Std	0.216	0.209	0.209
	Accuracy	Mean	0.749	**0.753**	0.752
		Std	0.215	0.214	0.212
Threefold Cross-validation	AUROC	Mean	**0.900**	0.899	0.899
		Std	0.078	0.081	0.077
	AUPRC	Mean	**0.952**	0.950	0.950
		Std	0.080	0.082	0.082
	Accuracy	Mean	**0.879**	0.878	**0.879**
		Std	0.086	0.088	0.085

Knowledge distillation models are represented as teacher → student. The left side of the arrow indicates the teacher model, and the right side of the arrow indicates the student model (ST: single-task learning model, MT: model of multi-task learning with similar tasks).

Significant values are in bold.

**Figure 3:**
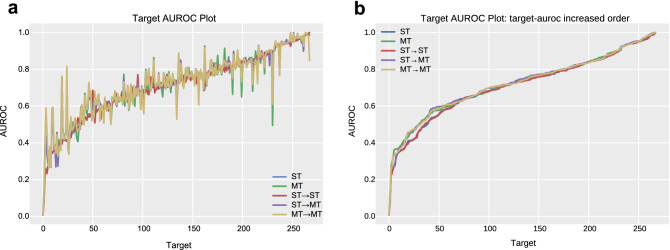
AUROC plot for the single-task model, multi-task model and knowledge distillation models. The blue line is a plot for the single-task learning model, and the green line is for the multi-task learning model. The red line is a plot for the single-task learning model distilled from the single-task learning model. The purple line plots the multi-task learning model distilled from the single-task learning model. The yellow line is a plot for the multi-task learning model distilled from the multi-task learning model. (**a)** The target order is the same in the five plots. (**b)** The target order of the five plots differs. The targets are ordered in target-AUROC increasing order.

Figure 3 shows the AUROC plots for all five models. As in the previous figures, the targets are sorted on the basis of their AUROCs from the single-task learning model in Fig. 3a. In addition, to compare the target-AUROC distribution among the five models, targets are arranged in target-AUROC ascending order in the right figure (Fig. 3b). Each plot thus has a different target order. Although the multi-task learning model distilled from the single-task learning model shows the highest performance, there is no distinct difference between the knowledge distillation models and their base models in the target-AUROC plots (Fig. 3a).

**Figure 4:**
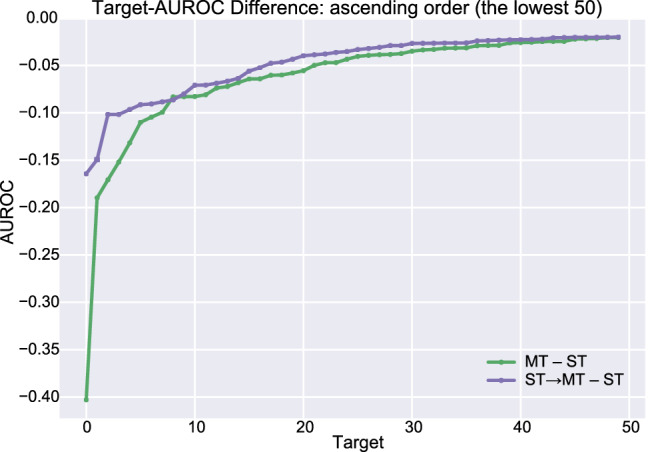
Target-AUROC difference from the single-task learning model. Targets are ordered in increasing order of AUROC difference. The targets with the lowest 50 AUROC differences are shown in this figure.

To determine why the multi-task learning model distilled from the single-task learning model shows the highest performance among the models, we plot the differences in target AUROC scores from the single-task learning model. When the target AUROC difference in a model is 0.1, the AUROC of target A resulting from this model is 0.1 higher than the AUROC of the same target resulting from the single-task learning model. As in the previous figure, the targets are ordered by increasing AUROC difference. In Fig. 4, the ST → MT model (purple line), which is the multi-task learning model distilled from the single-task learning model, shows a lower decrease in target AUROC than the multi-task learning model (green line). This result indicates that multi-task learning with knowledge distillation increases the performance of the model by minimizing the adverse effect of multi-task learning.

We also calculate the Pearson correlation coefficient and its significance for the change from the multi-task learning (MT–ST) and the change from the knowledge distillation (ST → MT–MT) for all targets (see Supplementary Fig. S2 online). The correlation coefficient is −0.501, and the significance is 1.893e−18, which clearly indicates that knowledge distillation from the single-task learning model to the multi-task learning model (ST → MT) improves more as the performance decreases from the multi-task learning.

### Case studies

We conduct a case study for a specific target protein to better explain our method. The results have shown that the multi-task learning with target clustering improves the average performance than the single-task learning and classic multi-task learning that trains a single model on all targets. Moreover, the held-out test dataset results have shown that applying knowledge distillation could improve performance by recovering the performance loss in individual tasks.

One of the examples that shows the effect of our multi-task learning method is the case of the norepinephrine transporter. The single task model that predicts the interaction of molecules to this target shows 0.8119 as its AUROC. After multi-task learning with all other targets, the AUROC for this target decreases to 0.772. The norepinephrine transporter is clustered with the serotonin transporter and dopamine transporter for clustered multi-task learning with similar targets. After multi-task learning with these two targets, the AUROC for this target is 0.779. Although this value is higher than the previous classic multi-task learning model, the value is still lower than the initial single-task learning model, showing a loss of performance. In this case, knowledge distillation recovers this performance reduction. After training the multi-task learning model with guidance from the single-task learning model, the AUROC value for prediction of interaction of molecules and this norepinephrine transporter increases to 0.843, showing that the performance is improved compared to the single-task learning model.

## Conclusions

We propose a method that improves molecular binding prediction by multi-task learning. Because multi-task learning may lead to performance degradation or a trade-off between individual task performance and average task performance, despite knowledge sharing, we introduce group selection and knowledge distillation to minimize these disadvantages. Our method results in the highest performance among four types of models—a single-task learning model, classic multi-task learning model and multi-task learning model—without knowledge distillation.

Further analysis shows that the performance gain of each task with multi-task learning increases as the initial performance of the task with single-task learning decreases. However, the gain from knowledge distillation is associated with the loss of performance after multi-task learning, thus suggesting that knowledge distillation helps the model recover its individual task performance.

## Methods

### Dataset preparation

The ChEMBL database is used for model training and testing. ChEMBL offers information for drug discovery, such as interactions of target proteins and molecules, and gene expression data^36,37^. After preprocessing according to^18^, ChEMBL version 23 is used for the model training and target clustering, whereas ChEMBL version 27 is used for the model testing. First, only single protein targets are collected. Second, only human proteins are used. Third, an assay with a confidence score of at least 8 is used.

Because classification tasks are performed, we set the labels of the molecules according to their activity values, following the threshold in^18^. We label the molecules as active toward a target when the target-molecule pair's activity value (IC50) is 104 nM or less. Otherwise, the molecule is labeled as inactive to the target. The target-molecule pairs are excluded when they have both active and inactive labels. We use extended connectivity fingerprints (ECFP)^38^ to represent the molecules for models, producing 2048 bit ECFP4 generated by RDKit^39^ from SMILES provided by ChEMBL. The molecules are excluded when RDKit does not produce their ECFP.

We divide the dataset from ChEMBL version 23 into a training dataset and a validation dataset (validation rate: 15%). This dataset is used in threefold cross-validation. Targets with fewer than 30 active and 30 inactive ligands are excluded from the training dataset. In addition, targets with fewer than three active ligands and three inactive ligands are excluded from the validation dataset. The dataset from ChEMBL version 27 is used for the test dataset to ensure that the test molecules differ from those from both the training and validation dataset. We filter out the targets with fewer than five new active ligands and five new inactive ligands. This dataset from ChEMBL version 27 is used as a held-out test dataset. After preprocessing, 374 target proteins and 523,539 molecules remain in the dataset. The training set has 370,024 molecules, the validation set has 65,279 molecules, and the test set has 88,236 molecules. Table 4 shows the description of dataset.

**Table 4:** Description of dataset size.

	Targets	Molecules		
Dataset size	374	Training	Validation	Test
		370,024	65,279	88,236

The number of targets is 374 for all datasets. Training dataset has 370,024 molecules. Validation dataset has 65,278. Test dataset has 88,236.

### Target clustering

We apply the similarity ensemble approach or SEA^32,33^ method on the ChEMBL version 23 dataset to cluster the target proteins. According to SEA, the similarity of each protein is determined, and then the distance between the target proteins is determined according to their similarity. We then apply hierarchical clustering. The SEA method from^32,33^ is used, and the following equations are from^33^. The SEA method builds the relationship between targets according to the similarity between their ligand sets. The raw score of the similarity between ligand sets is computed by summing all similarities for ligand pairs from two ligand sets if the similarity between this ligand pair exceeds the threshold value. The equation below explains the raw scores of the ligand set of target A and ligand set of target B. Because the raw score is affected by the size of the ligand sets, the influence of the ligand set size must be minimized. Here, we convert the raw score to a z-score, and then estimate the p-value as follows:

1 $$\begin{array}{l}z-score= \frac{Rawscore\left(A,B\right)-{F}_{mean}\left(s\right)}{{F}_{sd}\left(s\right)}\end{array}$$

2 $$\begin{array}{l}p-value= \left\{\begin{array}{l}1-{e}^{x\left(z\right)}\quad if \, \, z \le 28;\\ -x\left(z\right)- \frac{x{\left(z\right)}^{2}}{2}-\frac{x{\left(z\right)}^{3}}{6} \quad if\,\, z>28.\end{array}\right.\end{array}$$

3 $$\begin{array}{l}x\left(z\right)= -{e}^{-\frac{-z\pi }{\sqrt{6}-0.577215665}} \end{array}$$

where s denotes the product of the set A size and set B size. Therefore, it is the same as the number of ligand pairs produced from set A and set B. ${F}_{mean}(s)$ and ${F}_{sd}(s)$ are the expected raw score mean and the expected raw score standard deviation, respectively.

Before calculating the distance, the threshold should be determined. The procedure to decide the threshold value is as follows. First, 100 Tanimoto coefficient thresholds (TS) from 0.00 to 0.99 are prepared, with a step size of 0.01. Then the raw score for every TS is calculated and converted into a *z*-score. The TS value is selected from the chi-square test, to make the *z*-score distribution best fit the extreme value distribution. After determination of the TS value, the *z*-score from the selected TS is converted to the *p*-value, which is the significance of similarity of targets. In this work, the *p*-value from the SEA is used to decide the distance. Single linkage with average distance is used. For implementation, we use the Scikit-Learn library^40^. Then the appropriate threshold is chosen. We consider the size of the largest cluster and the number of single targets not included in any clusters. The number of whole clusters is also considered. For the clustering threshold, we use a threshold of 1e-50.

### Neural network

**Figure 5:**
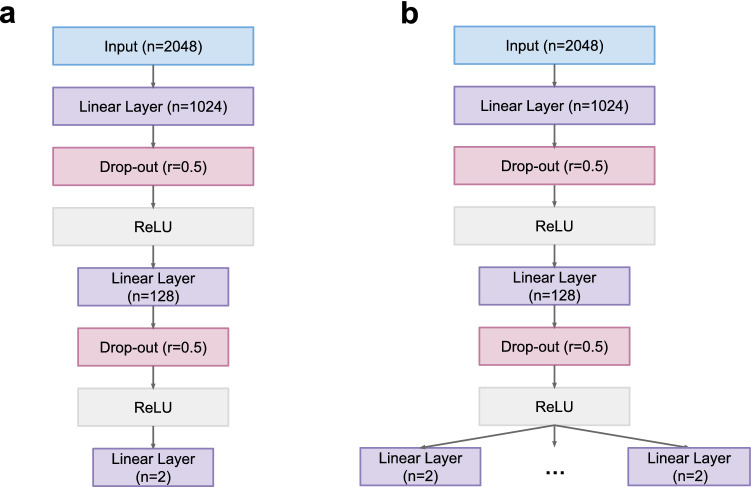
The architecture of models. (**a)** Single-task learning model. (**b)** Multi-task learning model.

Two types of models are used in this project. The first model is a single-task model, and the second is a multi-task model. Both models are based on a simple feed-forward neural network. These models use an ECFP vector of size 2048 as an input and generate predictions in the form of vectors of size 2 since they perform a binary classification for input ligand. The model predicts the interaction of given molecule to a single target (single-task model) or multiple targets (multi-task model). There are two hidden layers in the models. The first layer has 1024 nodes, and the second layer has 128 nodes. The first hidden layer in the multi-task learning model is a shared layer. All tasks share the weight of this layer. The second hidden layer is the model is a task-specific layer. The weight of this layer is not shared by tasks and differs for every task. The drop-out layer follows after every linear layer. The ReLU is used as an activation function. The Fig. 5 shows the architecture of the single-task learning model and multi-task learning model.

### Training details

In multi-task learning, we train the task-specific parts of the model individually. Not every input molecule has the records of the target proteins; consequently, the datasets of each target protein differ. Therefore, the shared layer is updated in every step, whereas the task-specific layer is updated when the model takes the input of the task’s dataset. The batch is composed of the input samples from the dataset of one task. The batch is fed into the model in random order. To prevent overfitting, early stopping is used in training, and the validation loss of each task is tracked during training. When overfitting appears in a task, the training of this task stops, and other tasks remaining in multi-task learning continue the training.

We applied the knowledge distillation from^31^. The labels for the student model use a weighted sum of the soft labels from the teacher model and the true labels of the input data. The following equation describes the student label (*y*) and the training loss (*Loss*) of the student model:

4 $$\begin{array}{l}y=\alpha {y}_{true}+\left(1-\alpha \right){y}_{teacher}\end{array}$$

5 $$\begin{array}{l}Loss={L}_{CE}\left({y}_{pred}, y\right)\end{array}$$

where ${y}_{true}$ and ${y}_{teacher}$ denote the true label of the input and the class probability distribution produced by the teacher model, respectively. α is a weight that increases from 0 to 1 through the training process. The cross-entropy loss ${L}_{CE}$ between the predicted output ${y}_{pred}$ and $y$ is used for the loss.

We test the model with many combinations of different hyperparameters. The maximum epoch and drop-out rate are fixed as 500 and 0.5. The batch size and learning rate are varied. The best hyperparameter combination is selected according to the validation loss. The hyperparameter combination resulting in minimal validation loss is considered the best hyperparameter set. For the single-task learning model, each task is trained alone, so the best hyperparameter combination is determined according to its validation loss. For the multi-task learning model, tasks in the same cluster are trained together. Thus, the best hyperparameters for these tasks are the same.

We use the area under the receiver operating characteristic curve (AUROC) as the major performance metric, because it is a widely used method for measuring the performance of classification models. We use the area under the receiver operating characteristic curve (AUROC) as the major performance metric because it is a widely used method for measuring the performance of classification models. We also use the area under the precision-recall curve (AUPRC) and accuracy to evaluate the performance. In this study, these evaluation metrics are measured for each target dataset to obtain the model’s performance for each target. They are called target-AUROC^18^, target-AUPRC, and target-accuracy. Because there are 268 targets, the model produces the target-AUROC set of 268 AUROC values, target-AUPRC set of 268 AUPRC values, and target-accuracy set of 268 accuracy values.

### Evaluation

We evaluated our models by two methods. The first method is threefold cross-validation and the second uses a held-out test dataset. In cross-validation, we use only the training and validation dataset and do not use the held-out test dataset. The dataset is split into three subsets. One subset is used as a test set, and the other two subsets are used as a training dataset. This process is repeated three times, so the model is tested on every subset. The model is also evaluated by the held-out test dataset. Since the test dataset is released later than the training dataset, it is composed of newly added molecules. It allows us to evaluate our model for unknown molecules.

## Supplementary Information

Supplementary Figures.

Supplementary Table S1.

## Data Availability

The datasets generated during and/or analysed during the current study are available at https://github.com/chaeyoungmoon/multi_task.
