# Supplementary material for: Prediction of drug–target interactions through multi-task learning
Source: Sci Rep. 2022 Oct 31;12:18323. doi: 10.1038/s41598-022-23203-y (PMC9622881; doi:10.1038/s41598-022-23203-y)

Supplementary Information:

Prediction of drug-target interactions through multi-task learning

Chaeyoung Moon and Dongsup Kim*

Department of Bio and Brain Engineering, Korea Advanced Institute of Science and Technology, Daejeon 34141, Republic of Korea

*Corresponding author (kds@kaist.ac.kr)

Supplementary Figure S1. Correlation between single-task target AUROC and target-AUROC difference. Target AUROC difference is calculated by subtraction of the target-AUROC of the single-task learning model from the target-AUROC of the multi-task learning model. The Pearson correlation coefficient is -0.34, and its p-value is 1.50e-08.

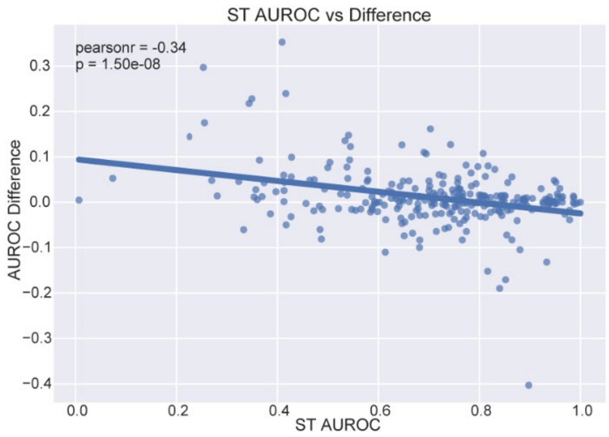

Supplementary Figure S2. Correlation between the target-AUROC change from multi-task learning and target-AUROC gain from knowledge distillation. The dots are AUROC gains of all 268 targets, and the line is the fitting model. The x-axis is the target-AUROC difference between the multi-task and single-task learning models (MT-ST). The y-axis is the target-AUROC difference between the multi-task learning model distilled from the single-task learning model and the multi-task learning model (ST→MT – MT).

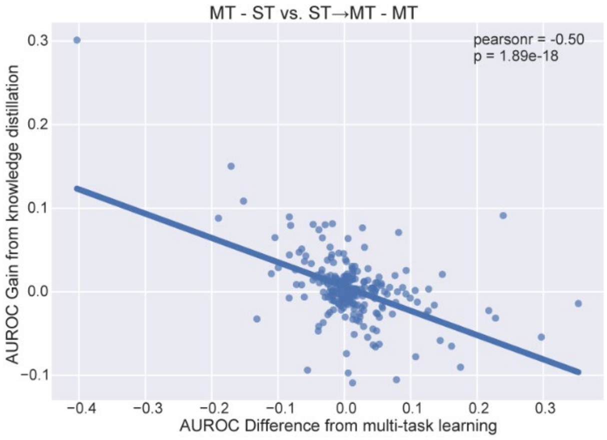

Supplement: Supplementary file 1 — Supplementary Figures. [file 41598_2022_23203_MOESM1_ESM.pdf]
